# Drugs acting on central nervous system (CNS) targets as leads for non-CNS targets

**DOI:** 10.12688/f1000research.3-40.v2

**Published:** 2014-03-21

**Authors:** Prashant S. Kharkar

**Affiliations:** 1 Department of Pharmaceutical Chemistry, SPP School of Pharmacy and Technology Management, SVKM’s NMIMS, Mumbai, 400056, India

## Abstract

Innovative drug discovery approaches are currently needed to rejuvenate the shrinking product pipelines of the pharmaceutical companies across the globe. Here a theme is presented – the use of central nervous system (CNS) drugs as leads for non-CNS targets. The approach is related to the use of existing drugs for new indications. Suitable chemical modifications of the CNS drugs abolish their CNS penetration. These novel analogs may then be screened for activity against non-CNS targets. Careful selection of the appropriate structural modifications remains the key to success.

## Commentary

The pharmaceutical industry worldwide is suffering from ‘productivity crisis’ ^1^. The number of new molecular entities (NMEs) launched annually has decreased significantly despite rising discovery and developmental expenses ^2^. Drug discovery researchers have started seeking approaches with higher probabilities of success. Drug repositioning, drug rescue and related strategies such as selective optimization of side activities (SOSA) are the front-runners ^3^. In May 2012, the US National Institutes of Health (NIH) launched ‘Discovering New Therapeutic Uses for Existing Molecules’, a collaborative program administered by the National Center for Advancing Translational Sciences (NCATS). Such an initiative emphasized the importance of lead discovery approaches based on existing drugs.

The design and development of drugs that cross the blood-brain barrier (BBB) and act at some target site(s) in the central nervous system (CNS) is a formidable task ^4^. In contrast, for peripherally-acting drugs, it is important to restrict their passage through the BBB in order to avoid unwanted CNS side effects. Several physicochemical and molecular properties of the CNS drugs differ from non-CNS oral drugs; the former have lower molecular weights, are more lipophilic, have a smaller topological polar surface area (TPSA), a fewer H-bond acceptors and donors and fewer rotatable bonds ^5^. There is a fine balance between the physicochemical properties of CNS and non-CNS drugs (see Table 1).

**Table 1.** Comparison between CNS and non-CNS drugs in terms of associated properties ^a, b^.

Molecular/Physicochemical property	CNS drugs	Non-CNS oral drugs
No. of H-bond acceptors	0–5 (2–3)	0–11 (3–4)
No. of H-bond donors	0–3 (0–1)	0–7 (2–3)
Molecular volume (Å ^3^)	800–1000 (740–970)	1000–1200
TPSA (Å ^2^)	<76 (25–60)	>80 (80–140)
No. of carboxylic acid groups ^c^	0	0–2
No. of basic amines	0–2	0–1
No. of polar H atoms	0–3	0–6
C-Het ratio (ratio of C atoms and non-C, non-H atoms	2.1–11	1.11–77.8
Molecular weight	100–450 (300–350)	100–650 (350–400)

^a^ Ghose *et al.* (2012) ^9^

^b^ The values in parentheses indicate the best ranges

^c^ Only 3–4% CNS drugs contain –COOH group whereas ~25% non-CNS oral drugs contain a –COOH group

During typical lead optimization cycles in the discovery phase, the lead molecules undergo several chemical modifications in order to improve their potency and pharmacokinetic (PK) properties, which usually lead to increased a) molecular weight, b) lipophilicity, c) molecular complexity, d) number of rotatable bonds, e) number of H-bond donors and acceptors, etc ^6^. In general, the drugs are more complex than the leads and exhibit higher values for the majority of the associated molecular properties listed above. Based on these findings, it can be hypothesized that ‘CNS drugs which are smaller and possess lower ranges of the aforementioned molecular properties make excellent starting points (as leads) for the development of non-CNS drugs’. Several aspects of this hypothesis are outlined in the discussion given below.

Majority of the CNS drugs are basic in nature ^4^. The presence of an ionizable functional group (mostly cationic) favors BBB penetration. Strong acids (pK _a_ < 4) and strong bases (pK _a_ > 10) are prohibited from crossing the BBB ^5^. Chemical modifications of the basic functional group (primary and secondary) to a neutral species (e.g., conversion of primary amine to a substituted urea or amide) may impede the entry of the NME into the CNS. Several physicochemical and molecular properties can then be tailor-made once suitable potency against a non-CNS target is found.

Another molecular property, TPSA, is crucial for BBB penetration. A TPSA cutoff of 90 Å ^2^ has been suggested for CNS drugs ^7^. Higher TPSA is likely to create hurdles in the passage of NMEs across the BBB. This can be achieved through the introduction of polar functional groups such as sulfonamide, carboxylic acid, substituted amides, etc., on the aromatic rings present in majority of the CNS drugs. Structural modifications leading to a higher TPSA will ultimately lead to an increased number of H-bond donors and/or acceptors and reduced lipophilicity. The cumulative effect is reduced CNS penetration.

The CNS drugs tend to have less molecular flexibility, lighter molecular weights and less molecular volume ^5^. Significant increases in these molecular properties may create obstacles in absorption following oral administration leading to reduced bioavailability, e.g., an increased number of rotatable bonds can result in increased hepatic metabolism of the drug ^8^. Nonetheless, the overall effects of an increase in the molecular weight and/or molecular flexibility on BBB penetration may depend on alterations in other properties such as molecular volume, solvent-accessible surface area (SASA), lipophilicity and TPSA. The distinction between the CNS and non-CNS oral drugs in terms of molecular and physicochemical properties is clearly evident from Table 1. Many of the common properties such as LogP, number of aromatic rings and ring assemblies and distribution coefficient [logD at pH 2 and pH 7.4) show little or no distinction between CNS and non-CNS oral drugs. Such properties were not included in Table 1.

In terms of toxicity, inhibition of the hERG channel by several CNS drugs (e.g., haloperidol) is a major concern. Many CNS drugs contain the hERG pharmacophore (aromatic rings and suitably placed cationic N) ^10^. Suitable chemical modifications of the CNS drugs such as attenuating the basicity of the cationic N and suitably placed aromatic substituents may lead to abolished hERG binding and associated adverse effects. Thus, conversion of a CNS drug into its non-CNS counterpart, according to the theme of this commentary, may lead to diminished hERG toxicity.

From the above discussion, it appears convincing that the CNS drugs can serve as suitable leads for non-CNS targets after appropriate structural modifications leading to considerable alterations in their property space. This leads to the question: what are the potential applications of such a strategy?

**Figure 1.**
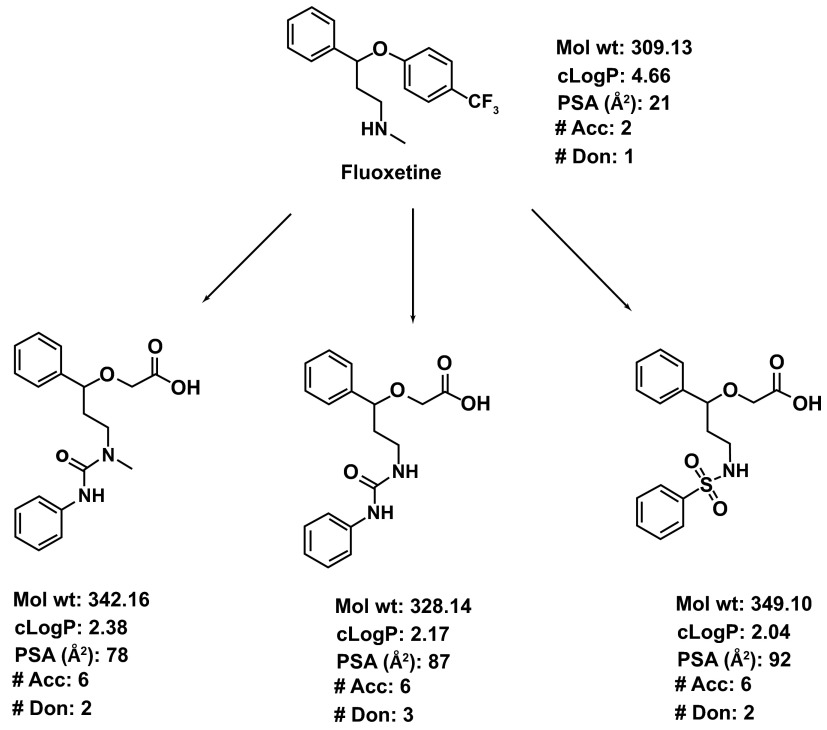
A theoretical illustration of the concept of using CNS drugs for non-CNS targets. Fluoxetine, an antidepressant agent, is used as a template. The synthesis of the analogs can be achieved by modifying the synthetic route of fluoxetine itself. The major structural modification includes abolishing the basicity of the secondary N in fluoxetine by converting it to urea or sulfonamide. Similarly, the 4-CF _3_Ph ring is replaced with an acetic acid side chain. The resultant structural modifications lead to increased PSA, decreased cLogP (calculated LogP) and an increased number of H-bond donors and acceptors.

Combinatorial libraries starting with CNS drugs can be designed *in silico* and then synthesized after selecting desirable substituents to introduce structural novelty (see Figure 1). These libraries may be unique in terms of structural and property space due to their origin from a known drug and thus may serve as a novel compound collection for high-throughput screening (HTS) campaigns. Once suitable hits are identified, every phase of non-clinical/investigational new drug work will need to be carried out with the lead molecules as normal, which may not reduce the development time due to a potentially different PK/PD (pharmacodynamic) profile of the newly designed entity. The development time may be reduced if a CNS drug is repurposed for a non-CNS indication. Several CNS drugs have already been repurposed for non-CNS indications, e.g., thalidomide (sedative/hypnotic to multiple myeloma and leprosy), chlorpromazine (antihistaminic to anticancer - inhibitor of the mitotic kinesin KSP/Eg5) ^11^, etc. Nonetheless, PK/PD, adverse reactions, toxicity and the clinical trials data obtained from the original drug may guide the design of such experiments for the non-CNS analogs. Similarly, the synthetic routes, bulk scale-up and the analytical methods may also be used for the novel non-CNS derivatives. The utility of any and every information about the CNS drug may vary from case to case. Since the original drug was never intended for a peripheral target, the intellectual property issues (novelty, non-obviousness) between the non-CNS analogs and the original drug analogs for the CNS target may be minimal.

Some functional groups convey particular therapeutic effects, e.g., acetic acid or related aliphatic acids as analgesic and anti-inflammatory agents (diclofenac, ibuprofen), sulfonamides as carbonic anhydrase (CA) inhibitors and as anti-bacterials (sulfisoxazole). As such introduction of these functional groups during chemical modifications of the CNS drugs may lead to a gain in potency for non-CNS targets such as CA. Careful selection of the chemical modifications aimed at the non-CNS target coupled to virtual screening of the designed analogs may potentially increase the rate of success of this approach.

In summary, the commentary outlines a novel approach for generating ‘interesting’ compound collections for lead discovery. Such an idea is of potential interest in times of pharmaceutical ‘productivity crisis’. The designed libraries can be tailor-made to suit the target requirements for potency and/or selectivity, in addition to pharmacokinetic and toxicity properties. Compared to the traditional lead discovery based on HTS (with or without virtual screening) of in-house or commercially available compound collections, the ‘intelligent’ (virtual or physical) libraries developed using the established CNS drugs may yield higher success rate (% of hits).

## Conclusion

The use of CNS drugs as a starting point for developing non-CNS leads seems interesting with reference to potentially altered molecular, PK/PD and/or toxicity properties. The combinatorial libraries based on the CNS drug scaffolds may perturb novel chemical space not accessed previously by the non-CNS small molecule drugs. Curious researchers interested in the above strategy may help in demonstrating its potential utility.
