# Supplementary material for: Drugs acting on central nervous system (CNS) targets as leads for non-CNS targets
Source: F1000Res. 2014 Mar 21;3:40. Originally published 2014 Feb 6. [Version 2] doi: 10.12688/f1000research.3-40.v2 (PMC3961999; doi:10.12688/f1000research.3-40.v2)
Supplement: Peer review history

# Peer review: Referee response for version 1

**Published:** 2014-03-07

**Authors:** Hariprasad Vankayalapati

**Affiliations:** 1 Huntsman Cancer Institute, University of Utah, Salt Lake City, UT, USA

This F1000Research article on “ *Drugs acting on central nervous system (CNS) targets as leads for non-CNS targets*" submitted by Kharkar is on a subject not uncommon to the CNS research community.

The following comments need to be addressed:

1. The author should state that several CNS agents, particularly multifunctional targets, are currently in clinical trial or have been approved for the treatment of non-CNS diseases despite the fact that they can cause inflammation and cancer. However, recently reported targeted CNS agents can bring an opportunity for repurposing some of the non-CNS targets as well. (Reference: http://www.ncats.nih.gov/research/reengineering/rescue-repurpose/rescue-repurpose.html).
   
2. Abstract: " *Out-of-the-box approaches are currently needed to replenish the souring *pipelines of pharmaceutical companies across the globe*" *this statement is arguable and needs to be revised.
   
3. Page 2 paragraph 9: this may not be true and needs to be revised completely to something like - " *Even the slightest modification to the physicochemical and physiological properties of the entity proposed as the non-CNS target of interest may alter its medicinal chemistry and tractability. One therefore needs to conduct every phase of non-clinical/IND work and may not reduce the development time due to the different PK and PD profile of the newly designed entity. However this is true only for repositioned drugs for new indications*".
   
4. Figure 1: The introduction of just a -COOH functional group as a strategy may not abolish the CNS activity completely (-COOH is negatively charged at the physiological pH). There is no evidence to support Figure 1 because reports show that at least 3−4% of CNS drugs have a carboxylic acid group. The author should therefore emphasize that this proposal is just theoretical as yet.  

The author also missed some important references that I feel need to be included to support this article:

a) Gosh *et al*., (2012) Knowledge-Based, Central Nervous System (CNS) Lead Selection and Lead Optimization for CNS Drug Discovery. *ACS Chem. Neurosci. *2012, 3, 50−68

b)  Palmer and Alavijeh (2012) Translational CNS medicines research. in *Drug Discov Today*.

I have read this submission. I believe that I have an appropriate level of expertise to confirm that it is of an acceptable scientific standard, however I have significant reservations, as outlined above.

# Peer review: Referee response for version 1

**Published:** 2014-02-26

**Authors:** Evans Coutinho

**Affiliations:** 1 Bombay College of Pharmacy, Santacruz, Mumbai, India

The author has proposed a method of using CNS drugs as leads and developing them into candidates for non-CNS target. The logic behind the approach is to tailor the attributes of a) molecular weight, b) lipophilicity, c) molecular complexity, d) number of rotatable bonds, e) number of H-bond donors and acceptors and f) polar surface area that are in consonance with values appropriate for non-CNS agents. He has illustrated his point with fluoxetine as an example.

 

This is an idea is worthwhile looking at, but the final test would be to see some real applications of the approach on some established CNS drugs and their activity on some non-CNS targets experimentally established.

I have read this submission. I believe that I have an appropriate level of expertise to confirm that it is of an acceptable scientific standard.

# Peer review: Referee response for version 1

**Published:** 2014-02-10

**Authors:** Vijay Gokhale

**Affiliations:** 1 College of Pharmacy, University of Arizona, Tucson, AZ, USA

This commentary describes a general approach for using CNS-active drugs as leads for non-CNS targets. As physicochemical properties for CNS-active drugs are more restrictive than for non-CNS drugs, it would appear that the CNS-active drugs could serve as a good leads. It is a well-written commentary discussing general approaches to modify properties of these lead molecules. The approach is also exemplified by use of fluoxetine as a lead and some suggested modifications of it.

 

One main problem with this approach is the challenge of removing or minimizing CNS activity of these leads for non-CNS targets since the activity on CNS will lead to side effects. It would be useful to include a table comparing desired value ranges for different physicochemical properties (molecular weight, log D, polar surface area, hydrogen bond donor) for CNS and non-CNS drugs. I am not aware if this approach has been used in the medicinal chemistry literature. But if such reports exist, it would be good to include those references in the commentary.

I have read this submission. I believe that I have an appropriate level of expertise to confirm that it is of an acceptable scientific standard, however I have significant reservations, as outlined above.
